# Supplementary material for: rcprd: An R package to simplify the extraction and processing of Clinical Practice Research Datalink (CPRD) data, and create analysis-ready datasets
Source: PLoS One. 2025 Aug 19;20(8):e0327229. doi: 10.1371/journal.pone.0327229 (PMC12364311; doi:10.1371/journal.pone.0327229)
Supplement: Attachment — Submitted filename: Comments PLos1.docx [file pone.0327229.s001.docx]

**Summary**

The authors introduce a new R package for simplifying data extraction and cleaning data for CPRD Aurum. Building on older but no longer maintained R packages, the authors present a software package storing the data as SQLite database. This is valuable tool for researchers using large electronic health records, given the time consuming data processing with pre-built functions to extract variables and with some included algorithms for data cleaning.

Overall, the paper and software package are very promising and useful but could be improved by clarifying some of the functionalities of the package and comparing the runtime to existing extraction approaches.

**Comments to the authors**

**General comments**

All worked examples work well. As a researcher who is regularly using CPRD, the main advantage of using a package as the one presented by the authors would be an improvement of runtime and less local RAM use when cleaning big patient dataset. I was wondering if the authors could present some comparisons of RAM use and runtime by extracting data from the SQLite database versus by looping through the CPRD Aurum raw data .txt files? As someone using datasets with up to 20mio patients, this would be the selling point of using the new R package.

**Introduction**

As this paper is targeted for an audience which has not used SQLite before, I’d suggest to add a half-sentence to explain how an SQLite database on a fixed storage device can align with the data storage requirements of CPRD usage (e.g., can be stored on a secure sever).

2.2 **Recommended process for extraction**

Cohort specification is often done with type 1 linkage of the data, i.e., using additional data from HES or the ONS mortality dataset. Could the authors clarify whether it is possible to use linked data for the cohort definition in Step 2.2?

**3.2.1 Add individual files to SQLite database using add_to_database**

When creating an SQLite database, it would be good to remind the reader that this should be done on a secure device as the following functions add raw CPRD data to the database.

**3.2.1 Add individual files to SQLite database using add_to_database**

Is it possible to store dates in a date format within the SQLite database? It can be sometimes very helpful to inspect the dataset for unrealistic date values which is more difficult if the dates are stored as a number.

**3.3.1 Functions for extracting common variable types**

Could the authors clarify some of the functionalities around the extraction functions:

- Is the index date of each function applied to the observation date or the enter date of a medical record?
- Do the function check for unrealistic dates, e.g., events recorded in 1899 or before the patient was born? These are common occurrences in CPRD data and should be removed from the final dataset.

Code lists are often include different categories of codes (e.g., a diabetes code list could contain codes for diagnoses, but also referrals, patient registries, examination results, etc). These different categories are often used in sensitivity analyses but also required if variables are defined based on algorithms, e.g., BMI, vaccinations, eGFR, etc.

I think it would make data extraction easier if the authors allowed the user to enter a data frame for the code list instead of a vector alone. This function could extract based on the medcodeid but would merge the other columns included in the code list data frame to the final output. I know that the same can be done by applying the current functions iteratively but I think it would improve the workflow of data extraction.

**Algorithms for data extraction**

BMI

I would like to flag that there are entries in CPRD in other units than cm/m and kg/stone (e.g., inches, feet, pounds, …). I would recommend the authors to double check that their algorithm includes all possible measuring units of height and weight used in CPRD.

Diabates status

How does the algorithm handle general diabetes codes, e.g., “diabetes mellitus”, “diabetic foot”, etc? In the current version it only allows to enter specific codes for either type 1 or type 2. However, as the generic codes are commonly used, I would recommend including them.
