# Supplementary material for: rcprd: An R package to simplify the extraction and processing of Clinical Practice Research Datalink (CPRD) data, and create analysis-ready datasets
Source: PLoS One. 2025 Aug 19;20(8):e0327229. doi: 10.1371/journal.pone.0327229 (PMC12364311; doi:10.1371/journal.pone.0327229)
Supplement: Attachment — Submitted filename: Peer review response R1 rcprd 20250403.docx [file pone.0327229.s003.docx]

Hello, many thanks to the editor and both reviewers for their time reviewing this manuscript. We have made considerable changes to the manuscript:

- Added a performance and scalability case study on synthetic CPRD data which is of comparable size to the real CPRD data. This case study is used to assess computational performance and quantify the benefit over the manual ‘for-loop’ approach.
- The ability to specify codelists through R data frames.
- Cleaning tools such as the ability to de-duplicate data, and the removal of dates before 1900 or prior to patient year of birth.
- Dates now returned in date formats after querying database.
- A new flow diagram and process which aligns with the process implemented by CPRD when cohort inclusion/exclusion criteria are dependent on linked data.
- Clarification over how diabetes codes and units of measurements have been handled.

We provide a comment-by-comment response below. Reviewer/editors comments are in black text, our response is in blue text, and changes to the manuscript in green text. Line numbers refer to the tracked changes manuscript.

**Editor comments**

Based on the reviewers’ assessments, the manuscript presents a valuable contribution to the field of EHR analysis by introducing the rcprd R package. However, some revisions are necessary before acceptance.

Required Revisions:

Performance and Scalability – The authors should include a comparison of runtime and RAM usage when extracting data from an SQLite database versus looping through raw .txt files. A practical case study with real CPRD data (if feasible) would further strengthen the manuscript.

We have added a comparison of runtime and RAM usage, which is implemented through a replicable performance and scalability case study. The case study uses synthetic CPRD Aurum data. A new section has been added in the manuscript (section 4) detailing the methods and results of this analysis. Code to implement this analysis is also available on GitHub.

Comparison with Existing Tools – A direct comparison with other relevant packages, such as rEHR and aurumpipeline, should be included to clarify the advantages of rcprd.

Regrettably, we do not think this suggestion is feasible given the packages do very different things. We have explained in detail to reviewer 2 why we think this will not be possible. This is the only suggestion that we have not been able to accommodate.

Data Quality and Extraction Functions – The authors should clarify how unrealistic date values and general diabetes codes are handled in the extraction functions. Additionally, confirming whether SQLite supports date formats would be useful.

We have added functionality for cleaning date values and changed the database query function to return dates in date formats. We have also explained our approach to dealing with general diabetes codes. This is all summarised in our response to reviewer 1.

Security Considerations – Explicitly state the importance of using a secure device when adding raw CPRD data to an SQLite database.

We have added in two statements to explicitly state the importance of using a secure device.

Line 82: The SQLite database must be created on a secure device or server which aligns with the data storage requirements of CPRD.

Line 268: It is imperative that when adding raw CPRD data to an SQLite database, that the SQLite database itself is stored in a secure environment which aligns with the data storage requirements of CPRD.

We have also made a number of other changes to incorporate reviewer suggestions, which are detailed below.

**Reviewer #1:** **Summary**
The authors introduce a new R package for simplifying data extraction and cleaning data for CPRD Aurum. Building on older but no longer maintained R packages, the authors present a software package storing the data as SQLite database. This is valuable tool for researchers using large electronic health records, given the time consuming data processing with pre-built functions to extract variables and with some included algorithms for data cleaning.

Overall, the paper and software package are very promising and useful but could be improved by clarifying some of the functionalities of the package and comparing the runtime to existing extraction approaches.

**Comments to the authors**

General comments
All worked examples work well. As a researcher who is regularly using CPRD, the main advantage of using a package as the one presented by the authors would be an improvement of runtime and less local RAM use when cleaning big patient dataset. I was wondering if the authors could present some comparisons of RAM use and runtime by extracting data from the SQLite database versus by looping through the CPRD Aurum raw data .txt files? As someone using datasets with up to 20mio patients, this would be the selling point of using the new R package.

We have added a comparison of runtime and RAM usage in a practical case study. Please see the new section 4 in the manuscript.

Please note, we have had to delete our raw CPRD Aurum data (common practice once intermediate datasets have been derived for data security reasons), therefore we have made use of the synthetic CPRD datasets for this: <https://www.cprd.com/synthetic-data>. The synthetic data has the same data structure as the real CPRD Aurum data, however is smaller in size. We have therefore duplicated these synthetic files over and over to create synthetic data which is of comparable size to the real CPRD Aurum data to compare runtime and RAM usage. Another benefit of using the synthetic CPRD data, is that if readers request the data themselves, they will be able to replicate the case study themselves. All code for the case study is available on our GitHub page: XXXX.

Please note, neither the synthetic data or real data can be shared freely, and access is tightly controlled by CPRD. It is therefore not possible to provide easy to access reproducible examples for users that utilise this data and showcase how to use the package. The main body of the paper therefore still utilises the same simulated data from the previous submission.

Finally, while the synthetic data we have created for the case study is still not as big as the data for 20 million patients, it’s on a similar order of magnitude, and the computational time for both methods scales linearly, so we can extrapolate the differences. This is discussed in the final paragraph of section 4. FYI, I work with similarly sized data (~ 20 million patients) and find the SQLite approach to work well. In fact, working with data this size was the motivator for taking up this approach.

Introduction
As this paper is targeted for an audience which has not used SQLite before, I’d suggest to add a half-sentence to explain how an SQLite database on a fixed storage device can align with the data storage requirements of CPRD usage (e.g., can be stored on a secure sever).

You are right about emphasizing data security when utilising rcprd. We have added the following sentence:

Line 82: The SQLite database must be created on a secure device or server which aligns with the data storage requirements of CPRD.

2.2 Recommended process for extraction
Cohort specification is often done with type 1 linkage of the data, i.e., using additional data from HES or the ONS mortality dataset. Could the authors clarify whether it is possible to use linked data for the cohort definition in Step 2.2?

Good question.

Yes, it is possible to use linked data for the cohort definition in step 2.2. Any dataset can be added to the sqlite database using the `add_to_database` function, and specifying a user-defined function (through the `extract_txt_func` argument) for reading in the text file and formatting the variables appropriately. There is also built in function for reading in the primary diagnosis HES file, which can be accessed through specifying `filetype = hes_primary`.

However, we don’t necessarily recommend doing this. The HES data is generally a lot smaller in size and there are little benefits to reading it into an SQLite database and querying this, in comparison to reading in the raw txt file(s) and querying them within R.

Re-reading the proposed steps after your question, we’ve realised it is more appropriate to have a step-by-step process that is in-keeping with the process of requesting of type 1 data, which is very often used for cohort selection. We have re-written the steps and put into a new Box, Box 1. This Box, a Flow diagram, replaces Figure 1.

Line 151: This process aligns with the process implemented by CPRD when cohort inclusion/exclusion criteria are dependent on linked data (see Q2 and step 4).

Finally, please note, because the new steps are contained in a text box, this hasn’t shown up as a tracked change. Not sure why this is the case.

3.2.1 Add individual files to SQLite database using add_to_database
When creating an SQLite database, it would be good to remind the reader that this should be done on a secure device as the following functions add raw CPRD data to the database.

Thanks this is an important suggestion. We have added the following reminder about data security at this point:

Line 268: It is imperative that when adding raw CPRD data to an SQLite database, that the SQLite database itself is stored in a secure environment which aligns with the data storage requirements of CPRD.

Also note, moving forwards, people will have to use the trusted research environment (CPRD Safe), so this will become less of an issue.

3.2.1 Add individual files to SQLite database using add_to_database
Is it possible to store dates in a date format within the SQLite database? It can be sometimes very helpful to inspect the dataset for unrealistic date values which is more difficult if the dates are stored as a number.

The short answer is no, you cannot store variables in date format in the SQLite database. They can be stored either as a number or a character string. When we initially read in the raw text files (using functions in file functions_read_txt.R), we do format them as date values. Upon writing into the SQLite database, it becomes a numeric variable, taking the underlying number that the date value is based on (reference date 1^st^ Jan 1970). When its’ read back in through a query, its read back in as a numeric.

I guess the point at which you would be inspecting the dateset would be after running a database query (i.e. using db_query)? I have changed the code so that db_query will format dates variables as dates. We now also use db_query to view the data, as opposed to RSQLite::dbGetQuery, to ensure that the date formatted values are what is viewed.

Please see the revised section 3.2.1, where all database queries return dates in date format.

Effectively, the date formats are now part of the natural flow of rcprd. The dates are still stored as numbers in the SQLite database, but the user will never come across this unless querying the data using RSQLite, which we don’t recommend or showcase.

3.3.1 Functions for extracting common variable types
Could the authors clarify some of the functionalities around the extraction functions:
• Is the index date of each function applied to the observation date or the enter date of a medical record?

Observation date and issue date. Have added the following text to make this clearer:

Line 666: Variables are calculated relative to the index date using the observation date (obsdate) in the observation file and the issue date (issuedate) in the drug issue file.

• Do the function check for unrealistic dates, e.g., events recorded in 1899 or before the patient was born? These are common occurrences in CPRD data and should be removed from the final dataset.

No they did not. This became very evident in these examples, given the dates are all simulated completely at random.

We have changed the `combine_query` and `combine_query_boolean` functions to remove unrealistic dates (any event before 1900, or before the patient was born). These functions are used internally in all the other functions to extract variables, so will have the desired impact.

Note this has had some minor changes on the results presented.

Code lists are often include different categories of codes (e.g., a diabetes code list could contain codes for diagnoses, but also referrals, patient registries, examination results, etc). These different categories are often used in sensitivity analyses but also required if variables are defined based on algorithms, e.g., BMI, vaccinations, eGFR, etc.
I think it would make data extraction easier if the authors allowed the user to enter a data frame for the code list instead of a vector alone. This function could extract based on the medcodeid but would merge the other columns included in the code list data frame to the final output. I know that the same can be done by applying the current functions iteratively but I think it would improve the workflow of data extraction.

Thanks for this suggestion. I am keen to improve workflow for others, but it’s difficult to predict/know in what ways other people work! We have made the suggested change which has impacted a number of functions in different ways.

The `db_query` function will now merge the output with the codelist dataframe if the codelist is specified using codelist_df. Please note for large queries, this may increase computation time.

For functions extract_time_until and extract_test_data, if the codelist is specified using codelist_df, the output will be merged with the codelist dataframe retaining the other columns from the codelist, as you’ve suggested. For extract_test_data, its the codes associated with the returned tests. For extract_time_until, the associated data is that of the first observation post index date. For all these variables, there will be NA data for individuals who did not have any data matching the codelist.

For all other variables (extract_ho, extract_bmi, extract_cholhdl_ratio, extract_diabetes, extract_impotence, extract_smoking, extract_test_data_var), the codelists can be specified through the codelist_df arguments, but the output will not be merged. This is because these variables are all based off algorithms which may require multiple observations to derive the output, and its unclear which codes to present. E.g. BMI may be derived from directly from a code for BMI, or from two codes of height and weight respectively. Extract_ho might have multiple matching codes in a patients history, again, its unclear which to present.

We have added text in a couple of places, and an example of doing this at the end of section 3.3.1:

Line 776: The codelists can also be specified through an R `data.frame` which must contain either a *medcodeid* or *prodcodeid* column. This may allow the user to run sensitivity analyses more easily if they would like to extract the same variable for different subgroups of the same codelist. For example: ….

Line 849: If the codelist is specified through an R `data.frame` with the `codelist_df` argument, the returned query will also contain the variables from the codelist `data.frame`.

Please let me know if I have misunderstood.

Algorithms for data extraction
BMI
I would like to flag that there are entries in CPRD in other units than cm/m and kg/stone (e.g., inches, feet, pounds, …). I would recommend the authors to double check that their algorithm includes all possible measuring units of height and weight used in CPRD.

You are correct.

We did not include these because we looked at the different unit of measurements for the height and weight records of a large cohort (approx 20 million, real data) of individuals aged 18 - 85 between 2005 – 2020. The results are reported in the article “Details on algorithms for extracting specific variables”. For weight, we found no measurements that were not in kg or stones (or NA). For height, we found no measurements that were not in centimetres or metres (or NA).

We therefore think the approach taken is appropriate.

Diabates status
How does the algorithm handle general diabetes codes, e.g., “diabetes mellitus”, “diabetic foot”, etc? In the current version it only allows to enter specific codes for either type 1 or type 2. However, as the generic codes are commonly used, I would recommend including them.

We assume generic codes refer to type 2, and that type 1 diabetes is only identified through a specific code. This is why if a user has a type 2 and type 1 code, the type 1 code takes precedent, because the type 2 code could just be a generic one.

We have clarified this in the text:

Line 841: For diabetes status, diabetes if often recorded with generic codes such as "diabetes mellitus", which does not specify which type. This is dealt with by assuming all generic codes refer to type 2 diabetes, unless that individual also has a specific type 1 diabetes code, in which case they will be determined to have type 1 diabetes as opposed to type 2.

Full details on the algorithm are available in the package documentation.

Line 846: The full details on extracting these variables are provided in the vignette titled [Details-on-algorithms-for-extracting-specific-variables](https://alexpate30.github.io/rcprd/articles/Details-on-algorithms-for-extracting-specific-variables.html).

We are aware that our algorithms for deriving specific variables are not perfect. There is a lack of convention for how to extract these things, and many researchers may do this in different ways. I was therefore somewhat hesitant to include these in the first place, as it may make the user-overconfident and stop thinking about these things critically themselves. However, we decided to include as it gives the user a number of examples of how to use the functions from section 3.3.3 (db_query, combine_query, etc) to write functions to derive specific variables. We have tried to make it clear that these algorithms should be adapted/changed based on the desired algorithm for defining the variable.

Line 848: “However, it is important to state, that the correct way to define a variable may change from study to study. Therefore when using these functions to extract variables, we encourage taking the time to ensure that the way the variable is extracted matches the definition in one’s study, and edit these functions and algorithms accordingly.”

If you have a specific suggestion about how to deal with generic diabetes codes that is different to how we approach it, please let us know, as we can use it to bolster this argument. We could use it as example of an alternative way to specify the diabetes variable, and then make a broader point about how there are multiple ways to define algorithms for extracting data, highlighting the importance of carefully considering this.

**Reviewer #2:** This manuscript introduces rcprd, an R package designed to streamline the extraction, processing, and querying of CPRD data. The package tackles key challenges inherent in working with large-scale EHR data by creating an SQLite database from raw .txt files and offering a suite of functions to extract both common variable types (e.g., history of a condition, time-to-event variables, test results) and specific variables (e.g., BMI, cholesterol/HDL ratio). The manuscript provides a detailed description of each function in the package. The work is timely and promises to significantly reduce redundant efforts in processing CPRD Aurum data, potentially benefiting a wide range of health data researchers. Overall, the manuscript is well-written, well-organized, and represents a significant contribution to the field of EHR analysis.

Thank you for your input and taking the time to read the submitted manuscript.

Major Comments:
Comparison to other packages: Does rEHR or any other package still remain a viable alternative for preparing analysis-ready data from CPRD Aurum data? The authors discussed the differences in approach rather than performance among rEHR, aurumpipeline, and rcprd. Would it be possible to provide a comparison with existing tools? A clear comparison would help highlight the distinct improvements offered by rcprd.
Regrettably, this is a very difficult suggestion to implement. You are right in that we focused on discussing the differences in approach rather than performance, this is because they can’t be used to do the same task, and therefore it is not possible to directly compare them. We will try and explain this in a little more detail here.

Primarily, rEHR is not compatible out of the box with CPRD Aurum data. Furthermore, it cannot be used to create datasets of the type returned in section 3.3.1 of the submitted manuscript without large amounts of extra coding from the user. It can be used to estimate incidence/prevalence rates, and apply case-control matching (things that rcprd is not designed to do), and for these tasks we direct readers there.

Aurumpipeline also cannot be used to create datasets of the type returned in section 3.3.1, without large amounts of coding from the user. It provides an interface to save CPRD Aurum dataset as parquet files, but points the user towards an Rstudio tutorial “Parquet using R” for how to query the database itself. It does little to simplify the process of querying the database and creating variables of the type that would form variables in an analysis-ready dataset. There are functions to “apply a codelist to flag conditions for each patient”, however this requires 4 steps from the user, as opposed to being combined into a single function. There are not functions to help build variables of any other type, or functions to help the user query the parquet database more easily, beyond a function to output all observations between two given dates.

Therefore, while we understand why a direct comparison between the packages sounds appropriate and natural, when you look at the detail of each package, they cannot be used to do the same thing. This is why we have chosen to highlight the different things they can do rather than attempt to compare them directly.

Scalability and practical Implementation: Although the demonstration using a simulated dataset effectively showcases the functionality, the example dataset is relatively small (containing only 12 patients and 8 .txt files). Could the authors expand on practical applications by including performance metrics or a case study using real CPRD data (if feasible) to further validate the package’s utility in real-world settings?

We have added a comparison of runtime and RAM usage in a practical case study. Please see the new section 4 in the manuscript.

Please note, we have had to delete our raw CPRD Aurum data (common practice once intermediate datasets have been derived for data security reasons), therefore we have made use of the synthetic CPRD datasets for this: <https://www.cprd.com/synthetic-data>. The synthetic data has the same data structure as the real CPRD Aurum data, however is smaller in size. We have therefore duplicated these synthetic files over and over to create synthetic data which is of comparable size to the real CPRD Aurum data to compare runtime and RAM usage. Another benefit of using the synthetic CPRD data, is that if readers request the data themselves, they will be able to replicate the case study themselves. All code for the case study is provided in the supplementary material and is available on the following GitHub page: <https://github.com/manchester-predictive-healthcare-group/CHI-CHARIOT/tree/main/project-3-rcprd>

Please note, the synthetic data cannot be shared freely as access is controlled by CPRD. It is therefore not possible to provide easy to access reproducible examples for users that utilise that data and showcase how to use the package. The main body of the paper therefore still utilises the same simulated data from the previous submission.

Finally, while the synthetic data we have created for the case study is still not as big as the data for 20 million patients, it’s on a similar order of magnitude, and the computational time for both methods scales linearly, so we can extrapolate the differences. This is discussed in the final paragraph of section 4. FYI, I work with similarly sized data (~ 20 million patients) and find the SQLite approach to work well. In fact, working with data this size was the motivator for taking up this approach.

Minor Points:
L165: The authors mention the naming convention for the EHR files. Is user input required for this, or do the CPRD Aurum data files already adhere to the naming convention described in Section 2.1?

From my personal experience, the raw files have always already been named this way upon receiving them from CPRD. I have some collaborators who have made use of the package, and the data they had was in the same naming format. However, these collaborators are all at UoM, and we are aware the naming convention may differ when CPRD delivers data to different institutions/companies, or even different users, and that this also may change over time institution to institution receiving the data.

We have therefore added the `str_match` functionality to both `add_to_database` and `cprd_extract` functions. This allows the user to specify the text string that will determine which raw files are added to the sqlite database, which will be of use if the naming conventions differ. For example, if your raw observation files were named “obs001”, “obs002”, etc, you could specify “str_match = obs” to add all files with this string to the sqlite database.

This is detailed in line 509: “The string to match on, function to read in the raw data, and the name of the table in the SQLite database, can be altered using the `str_match`, `extract_txt_func` and `table_name` arguments respectively. Note the use of `str_match` may be of particular importance if the naming convention of the raw data differs from what we have described above.”

L170: Instead of stating “(note, expecting CRAN submission in the next month),” could the authors provide the CRAN link if available? If not, would a specific expected availability date (e.g., year/month) be preferable?

Happy to say it has now been accepted on CRAN. We have added the link at the suggested place:

Line 173: After installing **rcprd**, either from CRAN (<https://CRAN.R-project.org/package=rcprd>) or the associated GitHub repository (<https://github.com/alexpate30/rcprd>)

Once all revisions have been agreed upon (which have resulted in changes to the package), we will push all the changes to CRAN as well.

L171: Since reference 12 is the GitHub link for the R package, might it be more straightforward to directly include the GitHub URL rather than citing it as a reference?

Have made the suggested change.

L432: When adding all relevant files at once, is there a checking step for duplicate records? Could this potentially pose an issue under certain circumstances?

This is a good point and we should have addressed duplicates more explicitly. They have been largely ignored because for most variables that we have functions to extract, duplicates will not impact the output (so in answer to your second question, predominately no, except in one case). However, we agree the package will be more comprehensive if we give the user the option to check for duplicates. We have therefore approached this in three ways.

Firstly, we have added a warning if there are duplicates of the raw files themselves (i.e. an entire file has been duplicated by mistake). See the `cprd_extract`function.

Secondly, we have added an argument to the `add_to_database` and `cprd_extract` functions (`rm_duplicates`) to deduplicate the data before adding into the SQLite database. Note, we do not make this happen by default, as it does increase computational time, and for most variables their derivation will remain unchanged if duplicates values are present.

Thirdly, we have added an argument in the database query function `db_query` (`rm_duplicates`) to deduplicate after querying the SQLite database. Again, we do not make this happen by default as it does increase computation time, and `db_query` is used internally in a lot of other functions to derive variables which would be unchanged in the presence of duplicate values.

This functionality will be evident to users through the package documentation. We have also added the following text:

Line 512: The argument rm_duplicates = TRUE can be specified to de-duplicate records before adding into the SQLite database. This will increase computation time, and the derivation of many variables will not be effected by having duplicate records, so consider carefully whether it's necessary to apply this step or not. There will also be the opportunity to de-duplicate the records later on when querying the data.

Line 858: Setting rm_duplicates = TRUE will de-duplicate the output.

We now talk through each of the functions to verify/explain that they will not be impacted by duplicates, and therefore why we haven’t made this the default in the aforementioned changes.

‘Extract_ho’, returns a 0/1 vector depending on whether each individual has at least one observation with relevant code between. Any duplicate records will therefore not affect the output.

‘Extract_time_until’ returns the time until first code of interest or censoring, and an event/censoring indicator. Any duplicate records will therefore not affect the output.

‘Extract_test_recent’ returns the most recent test result that meets specified criteria. Any duplicate records will therefore not affect the output.

‘Extract_smoking’, ‘extract_bmi’, ‘extract_cholhdl_ratio’ all return the most recent value. Any duplicate records will therefore not affect the output.

‘Extract_diabetes’ returns diabetes status based on any record prior to the index date. Any duplicate records will therefore not affect the output.

‘Extract_test_data_var’ returns the standard deviation of all test data values over a specified time period relative to an index date. This WILL be impacted by duplicate records. We have a made a change so that duplicate records on the same day are ignored by default.

L581: There appears to be a typo: “ust” -> “must” ?.

Thanks, corrected.

Other minor change:

- We have changed the codelist used to showcase the db_query functionality in section 3.3.3, to something that returns more than one observation per patient.
- Changed `db_query` to `my_db_query` when it’s an object, as to not be confused with the function `db_query`.
- Changed `codelist` to `my_codelist_vector` when it’s an object, as to not be confused with the function argument `codelist`.
